# *Tequila*, the Serine Protease, Is Involved in Sleep-Dependent Memory Consolidation in *Drosophila*

**DOI:** 10.1523/ENEURO.0566-24.2025

**Published:** 2025-08-27

**Authors:** Aishwarya Segu, Shrutika Sansaria, Nisha N. Kannan

**Affiliations:** Chronobiology Laboratory, School of Biology, Indian Institute of Science Education and Research (IISER), Thiruvananthapuram, Kerala 695551, India

**Keywords:** daytime sleep, *Drosophila*, sleep fragmentation, sleep-dependent memory, *tequila*

## Abstract

Sleep is a vital physiological phenomenon observed among almost all organisms. Although its exact purpose remains elusive, sleep has been linked to memory consolidation. In our present study, we investigated the role of sleep quality on sleep-dependent memory consolidation. Previous studies have shown that *tequila*, a serine protease, affects long-term memory (LTM) consolidation in flies. In the present study, we identified that the hypomorphic mutation in the *tequila* gene (*tequila^f01792^*) leads to increased daytime sleep fragmentation at a very early age in male flies. Intrigued by this observation, we delved into further understanding the role of *tequila* in sleep-dependent memory consolidation by manipulating sleep duration using pharmacological methods such as GABA-A agonist. Inducing sleep using GABA-A agonist resulted in improved sleep during the day. This further led to a significant improvement in the LTM of these flies when compared with the vehicle-treated flies. In conclusion, daytime-dependent sleep fragmentation is possibly one of the reasons behind the LTM deficit observed in *tequila^f01792^* flies. Furthermore, we observed that these flies had disturbed sleep only during the daytime, whereas in the night the flies had increased sleep duration. The increased sleep fragmentation during the daytime possibly leads to memory impairment and rescuing sleep fragmentation partially rescues memory consolidation. These outcomes suggest that the *tequila* gene is involved in sleep-dependent memory consolidation.

## Significance Statement

Sleep is widely conserved across animal kingdom, although its duration varies between species. While the exact need for sleep remains unclear, its positive influence on memory is well documented. Studies have shown the interplay between sleep and memory, where learning enhances sleep drive and subsequently adequate sleep aids memory formation. However, for the first time our study highlights the importance of sleep quality in memory consolidation. The *tequila^f01792^* mutant flies, although showed no effect on sleep duration, had fragmented day sleep, which ultimately plays a significant role in memory consolidation. Thus, it is not just the quantity of sleep, but also the quality and consolidation of sleep are important for memory consolidation.

## Introduction

Memory formation is an intricate biological process largely driven by sensory perceptions, leading to neuronal encoding through various molecular and cellular mechanisms (Margulies et al., 2005; Marquand et al., 2023). Multiple internal factors like sleep quality, synaptic plasticity, and gene expression can influence the processes of learning and memory consolidation in organisms (Marquand et al., 2023). Sleep is a periodic quiescent phase observed in most organisms ranging from simple jellyfish to complex humans (Miyazaki et al., 2017; Keene and Duboue, 2018). It is crucial for many physiological and cognitive functions such as maintaining homeostasis, regulating metabolism and memory consolidation (Hendricks et al., 2000; Keene et al., 2010; Dissel, 2020). One of the hypotheses explaining how sleep contributes to consolidating memory is the synaptic homeostasis hypothesis (SHY). SHY states that synaptic downscaling, which occurs during sleep, contributes to memory consolidation by maintaining synaptic homeostasis and strengthening specific connections through long-term potentiation (Cirelli, 2013; Tononi and Cirelli, 2014).

Previous studies across multiple organisms including humans, mammals, and invertebrates have reinforced the importance of sleep in memory consolidation (Stickgold, 2005; Donlea et al., 2011; Dzierzewski et al., 2018; Chouhan et al., 2021; Lei et al., 2022; Marquand et al., 2023). Studies in *Drosophila* showed that sleep deprivation can significantly impair the performance index (PI) in various memory and learning assays (Bushey et al., 2007). Also, in the *Drosophila* model of Alzheimer's disease, increasing the sleep duration was shown to reverse cognitive deficits (Dissel et al., 2017). Furthermore, genes *dunce* and *rutabaga*, essential in memory formation and consolidation, are also crucial for regulating sleep (Levin et al., 1992; Dissel et al., 2015; Kirszenblat et al., 2018; Scheunemann et al., 2018). Deletion of these genes is known to cause a reduction in sleep duration, and by inducing sleep, short-term memory (STM) was partially recovered in these flies (Dissel et al., 2015; Kirszenblat et al., 2018). The key genetic insights into sleep-dependent memory consolidation have come from these studies done on *dunce* and *rutabaga* mutants. Despite the significance of sleep in memory, the evident genetic insights into sleep-dependent memory consolidation are still largely lacking. In our present study, we uncover the role of sleep quality in memory consolidation associated with *tequila*, one of the genes involved in long-term memory (LTM) formation. To do this, we used *Drosophila* as our model organism owing to its well-versed genetic tools and similarity to mammals in sleep behavior and memory consolidation (Duffy, 2002; del Valle Rodríguez et al., 2011; Hales et al., 2015; Beckwith and French, 2019).

*tequila* gene encodes a multidomain serine protease in *Drosophila*. It is shown that hypomorphic mutation in the *tequila* gene (*tequila^f01792^*) leads to LTM defects in flies, indicating its potential importance in synaptic plasticity (Didelot et al., 2006), and is also associated with an increased life span by modulating *Drosophila* insulin-like signaling (Huang et al., 2015). Studies also showed that *tequila* gene may influence memory formation in a nutrient-dependent manner (Plaçais and Preat, 2013).

Our study explored whether the *tequila* gene influences sleep and if so, its role in sleep-dependent LTM consolidation in *Drosophila*. We show that *tequila^f01792^* mutants do have a role in sleep consolidation and the functional copy of the gene is important for LTM. In flies, the deletion of the *tequila* gene leads to increased sleep fragmentation. Interestingly, these flies exhibit increased sleep fragmentation only during the light phase of the 12 h light/dark cycles (LD). Finally, the improvement of sleep quality in *tequila^f01792^* flies was indeed enough to significantly improve the LTM defect observed in these flies. Thus, we conclude that *tequila* plays a role in sleep-dependent memory consolidation.

## Materials and Methods

### Fly husbandry and maintenance

All the fly lines used in the experiments were grown in normal cornmeal-dextrose-agar medium. The fly lines used in the study are listed in Table 1. They were maintained at 25°C, with 65–70% humidity under LD in a cooled incubator (Percival Scientific). *tequila^f01792^* flies were backcrossed with *w^1118^* flies for seven generations to remove the genetic background effects. To do genetic manipulations of the *tequila* gene, we used the bipartite UAS-Gal4 system. The details of the lines are given in Table 1. The Gal4 and UAS lines were crossed with *w^1118^*, respectively, as genetic control lines.

**Table 1.** List of fly lines used in the study

Fly lines	Description	Source
*w^1118^*	*White* gene mutant, genetic background control	BDSC-5905
*W^1118^; PBac{WH}tequila^f01792^* (Backcrossed)	*tequila* gene hypomorphic mutant	BDSC-18473
*w^1118^;MB247-Gal4;* (backcrossed)	Mushroom body (MB) specific Gal4	Dr. Jishy Varghese (IISER TVM)
*y1 v1; P{TRiP.GL01328}attP40* (backcrossed with TRiP control)	*tequila*RNAi	BDSC-41896

### Sleep recording and analysis

Drosophila Activity Monitor (Model DAM2 for 5 mm tube) system was used for recording sleep and locomotor activity. Briefly explaining the process, individual flies were loaded into locomotor activity glass tubes containing food on one end and a cotton plug on the other end. The glass tubes containing flies were placed in a DAM2 monitor which contains an IR beam generator. Sleep was recorded for 5 consecutive days under LD > 5 min (min) of inactivity was considered an episode of sleep (Driscoll et al., 2019). The age and the sex of the fly were determined according to the experimental requirements. A total of 25–32 flies were used for the sleep recording under each condition. Sleep parameters, including total sleep duration, sleep episode duration, sleep episode number, and sleep profile, were analyzed using SCAMP (Vecsey et al., 2024). Sleep episode numbers were further classified into light (5 to <10 min sleep episode) and deep sleep episodes (>10 min) to better understand sleep fragmentation (Tainton-Heap et al., 2021). To do this a custom code script was written using Python 3.10.14 and the sleep data was analyzed. The codes used in the study are freely available on GitHub (https://github.com/Aishwarya-segu/Light-and-deep-sleep-fragmentation-analysis-in-Drosophila.git).

### Appetitive associative olfactory learning using Y-maze

A total of 60–75 male flies, aged 2–5 d, were trained with two aversive odors (conditioned stimulus CS), namely, 1-octanol (OCT) and 4-methylcyclohexanol (MCH). Odor associated with 2 M sucrose unconditioned stimulus (US) is termed as CS+ and the odor not associated with US is termed as CS− from here on. The protocol was adapted and modified from Mohandasan et al. (2022). Briefly explaining, the flies were starved in 1% agar for 12 h prior to training to induce hunger. The odors used were of the concentration 1:1,000 (odor:mineral oil). Post starvation, odor cups in the training vials were loaded with 20 µl of odor-filled filter paper and saturated for 20 min before each training. Each group of flies were then trained with CS− odor for 2 min followed by 5 min of rest in 1% agar vials followed by CS+ odor for 2 min. For each experiment, two sets of flies were trained with each of the odors associated with US (CS+) to avoid naive odor preferences. Posttraining flies were transferred to 1% agar vials before testing. STMs of flies were tested 3 h posttraining, and LTMs of flies were tested 24 h posttraining using a Y-maze. During the testing, flies were loaded onto the Y-maze and allowed to acclimate for 60 s. Post acclimatization, the flies were tapped to the bottom of the loading vial and allowed a 90 s decision period to choose between the odors present on each arm of the Y-maze. Female flies were not included in the experiment due to the challenges with egg-laying and reduced movement in the Y-maze. The flies undergo 36 h of starvation during the testing period, leading to eggs hatching and media liquefying. Due to this the females died in the vials. A total of 5–15 such biological replicates each containing 60–75 male flies were used, and performance index of flies was calculated using the formula given below (Walkinshaw et al., 2015):

$$\begin{array}{c} \mathrm{Half}\;\mathrm{Performance}\;\mathrm{index}=\frac{[(\mathrm{no}.\;\mathrm{of}\;\mathrm{flies}\;\mathrm{in}\;\mathrm{CS}+\mathrm{arm})-(\mathrm{no}.\;\mathrm{of}\;\mathrm{flies}\;\mathrm{in}\;\mathrm{CS}-\mathrm{arm})]}{\mathrm{Total}\;\mathrm{flies}\;\mathrm{who}\;\mathrm{made}\;a\;\mathrm{choice}} \end{array}$$

$$\mathrm{Performance}\;\mathrm{Index}\;(\mathrm{PI})=\frac{\left(\mathrm{Half}\;\mathrm{PI}\;1(\mathrm{odor}\;A)+(\mathrm{Half}\;\mathrm{PI}\;2(\mathrm{odor}\;B\right)}{2},$$

where PI < 0 indicates there was a negative choice of the odor, PI = 0 indicates equal choices of odor was made, and PI > 0 there was a positive choice made.

### Gaboxadol hydrochloride/4,5,6,7-tetrahydroisoxazole(5,4-c)pyridin-3-ol (THIP) drug treatment

#### Sleep recording and THIP treatment

Two-day-old male flies were individually transferred into glass tubes and loaded into DAM2 monitors, and their baseline sleep was recorded for 2 consecutive days. Post 2 d of baseline sleep recording, flies were flipped to glass tubes containing cornmeal-dextrose media with either 0.1 mg/ml of THIP or vehicle (MilliQ), and their sleep was recorded for 24 h. A total of 25–32 flies were used for recording sleep in each condition. Sleep analysis was performed as described earlier by using SCAMP.

#### Y-maze assay followed by THIP treatment

A total of 60–75 male flies, aged 2–5 d, were treated with either cornmeal-dextrose media containing 0.1 mg/ml of THIP or vehicle control (MilliQ) in glass vials for 36 h. Post-drug ingestion, the flies were transferred to 1% agar containing 0.1 mg/ml of THIP for 12 h prior to odor-associated training. Training and memory testing was performed as described earlier. Six to seven such biological replicates each containing 60–75 flies were used for the experiment. Different sets of flies were used for both the sleep recording and the Y-maze assay involving THIP treatment.

#### Sleep deprivation and memory assay

To deviate the effects of drug ingestion from sleep-induced memory, flies were sleep deprived using a mechanical shaker during the THIP treatment. To do this, 60–80 male flies were transferred to vials either containing THIP or vehicle control and were placed on a mechanical shaker for 48 h. Another set of flies with all the conditions identical except for mechanical deprivation was used as sleep nondeprived control. Three such biological replicates were performed. Post-drug treatment flies underwent 12 h of starvation with THIP, followed by training, and memory testing as described previously.

### Statistical analysis

All the data was tested for normality using the Kolmogorov–Smirnov test (with sample size 3 and less than 5 Shapiro–Wilk test was used). Datasets having normal distribution were analyzed for statistical significance using parametric tests, while the others were analyzed using nonparametric tests. Differences in variance between the two groups were tested using an unpaired Student's *t* test or Mann–Whitney test based on the distribution of the data. For multivariate analysis, ANOVA/two-way ANOVA or Kruskal–Wallis test was used based on the distribution of the data. All the statistical operations were performed and the graphs were plotted using GraphPad Prism 10.2.

## Results

### Mutation of *tequila* gene alters sleep quality of flies

**Figure 1.**
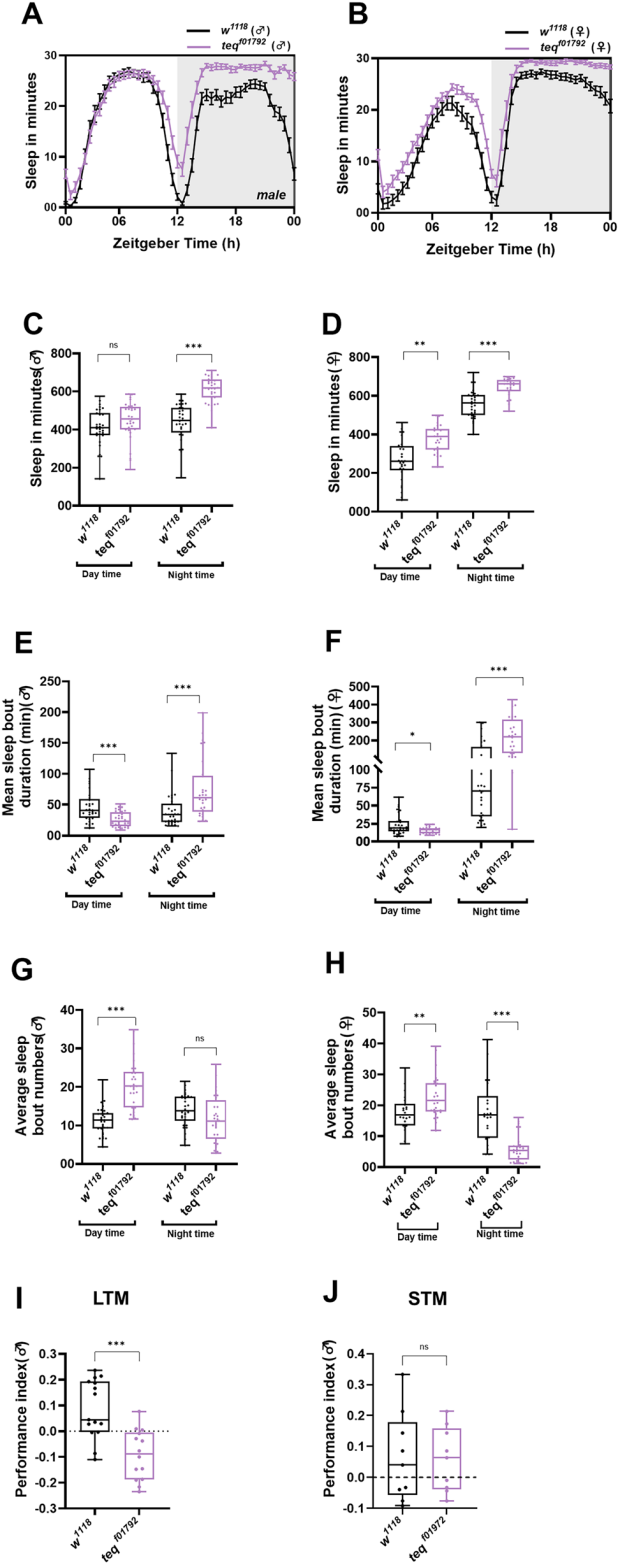
Mutation of *tequila* gene alters sleep quality of flies. ***A***, ***B***, Sleep in minutes for every 30 min over a period of 24 h under LD for 2-d-old *w^1118^* and *tequila^f01792^* flies. ***A*** and ***B*** depict the sleep profile of male and female flies, respectively. ***C***, Quantified day and night sleep in minutes of 2-d-old male *w^1118^* and *tequila^f01792^* flies. ***D***, Quantified day and night sleep in minutes of 2-d-old female *w^1118^* and *tequila^f01792^* flies. ***E***, ***F***, Quantified mean sleep bout duration in min for 2-d-old male and female *w^1118^* and *tequila^f01792^* flies, respectively. ***G***, ***H***, Average sleep bout number for 2-d-old male and female *w^1118^* and *tequila^f01792^* flies, respectively. ***I***, LTM performance index using pavlovian appetitive olfactory memory of *w^1118^* and *tequila^f01792^* flies. A significant decrease in memory is observed in *tequila^f01792^* flies. ***J***, STM performance index using operant appetitive olfactory memory of *w^1118^* and *tequila^f01792^* flies. Extended Data Figure 1-1 supports this figure.

*tequila*, a serine protease, is shown to be essential for LTM formation in flies (Didelot et al., 2006); further, it also plays a role in the life span (Huang et al., 2015). In our study, we aimed to elucidate the effects of the *tequila* gene on sleep. To do this, we used 2-d-old male and female hypomorphic mutants of *tequila* (*tequila^f01792^*) backcrossed with their genetic control *w^1118^* and their sleep was recorded. *tequila^f01792^* mutant male and female flies showed an increase in sleep compared with the control *w^1118^* flies (Fig. 1*A*,*B*). We quantified their sleep and observed an increase in sleep only during the nighttime in males (Fig. 1*C*; *w^1118^* vs *tequila^f01792^ t*_(61)_ = 8.135, *p* < 0.001, unpaired *t* test). However, in female flies, apart from an increase in night sleep, we also observed an increase in day sleep [Fig. 1*D*; *w^1118^* (daytime) vs *tequila^f01792^* (daytime) *t*_(53)_ = 3.319, *p* = 0.002, unpaired *t* test; *w^1118^* (nighttime) vs *tequila^f01792^* (nighttime) *p* < 0.001, Mann–Whitney test]. Sleep depth, regulated by the homeostatic pathway, is determined by two parameters—amount of sleep and sleep quality. The quality of sleep is defined by the length of each sleep bout and the number of sleep bouts. Lesser the number of bouts and the longer the duration of each bout, the better their sleep quality is. We further analyzed the sleep quality in *tequila^f01792^* mutant flies. Interestingly, mean sleep bout duration in *tequila^f01792^* mutant in both male and female flies is decreased during the daytime and increased during the nighttime [Fig. 1*E*,*F*; mean sleep bout duration; *w*^*1118(*♂*)*^ (daytime) vs *tequila*^*f01792 (*♂*)*^ *t*_(59)_ = 4.213, *p* < 0.001, unpaired *t* test; *w*^*1118 (*♂*)*^ (nighttime) vs *tequila*^*f01792 (*♂*)*^ *p* < 0.001, Mann–Whitney test; *w*^*1118(*♀*)*^ (daytime) vs *tequila*^*f01792 (*♀*)*^ *p* = 0.03, Mann–Whitney test; *w*^*1118 (*♀*)*^ (nighttime) vs *tequila*^*f01792 (*♀*)*^ *p* < 0.001, Mann–Whitney test]. In accordance, an increase in average sleep bout numbers during the day was observed in both males and females (Fig. 1*G*,*H*; *w*^*1118(*♂*)*^ vs *tequila*^*f01792 (*♂*)*^ *t*_(61)_ = 7.041, *p* < 0.001; *w*^*1118(*♀*)*^ vs *tequila*^*f01792 (*♀*)*^ *t*_(52)_ = 10.97, *p* < 0.001, unpaired *t* test). But, unlike sleep bout duration, we did not observe any significant decrease in the sleep bout numbers during the night in male flies, but the female flies showed a significant decrease in average sleep bout numbers (Fig. 1*G*,*H*; *w*^*1118 (*♀*)*^ vs *tequila*^*f01792 (*♀*)*^ *t*_(51)_ = 4.652, *p* < 0.001, unpaired *t* test).

10.1523/ENEURO.0566-24.2025.f1-1 Figure 1-1 *tequila ^f01792^* flies female flies display increased sleep bout numbers and increased bout duration. (A) Day and nighttime light and deep sleep bout numbers of 2-day-old *w^1118^* and *tequila ^f01792^* female flies. An increase in the light and deep sleep bout numbers during the day-time was observed. (B) Day and nighttime light and deep sleep bout numbers of 2-day-old *w^1118^* and *tequila ^f01792^* male flies. (C) Quantified total locomotor activity of 2-day old male and female *w^1118^* and *tequila ^f01792^* flies respectively. A decrease in activity was observed in *tequila ^f01792^* flies. Download Figure 1-1, DOCX file.

In female flies, we observed an increase in both daytime sleep and number of sleep bouts. This was surprising, and to further verify this we divided the sleep bouts based on their duration. Light sleep with episodes <10 min and deep sleep episodes as >10 min. In females an increase was observed in both daytime light and deep sleep episode numbers (Extended Data Fig. 1-1*A*) resulting in increased sleep and sleep bout numbers during the daytime. Whereas, in male flies during the daytime, an increase in the light sleep bouts and decrease in the deep sleep bouts was observed leading to an overall increase in daytime sleep bout numbers without any significant difference in the day sleep (Extended Data Fig. 1-1*B*). However, during the nighttime, both male and female *tequila^f01792^* flies displayed decreased deep sleep bout numbers when compared with the control. Furthermore, in female flies we observed a decrease in nighttime light sleep bout numbers as well (Extended Data Fig. 1-1*A,B*). In female flies, during the daytime, we observed not only increased sleep fragmentation but also an increase in sleep. These results suggest that reduced expression level of *tequila* gene leads to fragmented sleep during the day and an increase in sleep duration at night as a mode of sleep consolidation particularly in males.

To determine whether the changes in locomotor activity are associated with the increased sleep observed in mutant *tequila^f01792^* flies, we measured the total locomotor activity in 2-d-old male and female *tequila^f01792^* flies. Mutation in the *tequila* gene resulted in decreased locomotor activity in flies when compared with the control (Extended Data Fig. 1-1*C*)

Previously, *tequila^f01792^* flies have been shown to impair LTM formation induced by aversive olfactory conditioning. We further assessed whether *tequila* is necessary for LTM consolidation induced by pavlovian appetitive olfactory conditioning and a significant decrease in LTM was observed (Fig. 1*I*; *w*^*1118(*♂*)*^ vs *tequila*^*f01792 (*♂*)*^ *t*_(28)_ = 3.599, *p* = 0.001, unpaired *t* test). We also measured their 3 h STM, but no impairment in STM was observed (Fig. 1*J*). Thus, our study demonstrates for the first time that *tequila* is essential for LTM consolidation induced by appetitive olfactory conditioning.

### Mutation of the *tequila* gene leads to an increase in daytime sleep fragmentation at a very early age in male flies

**Figure 2.**
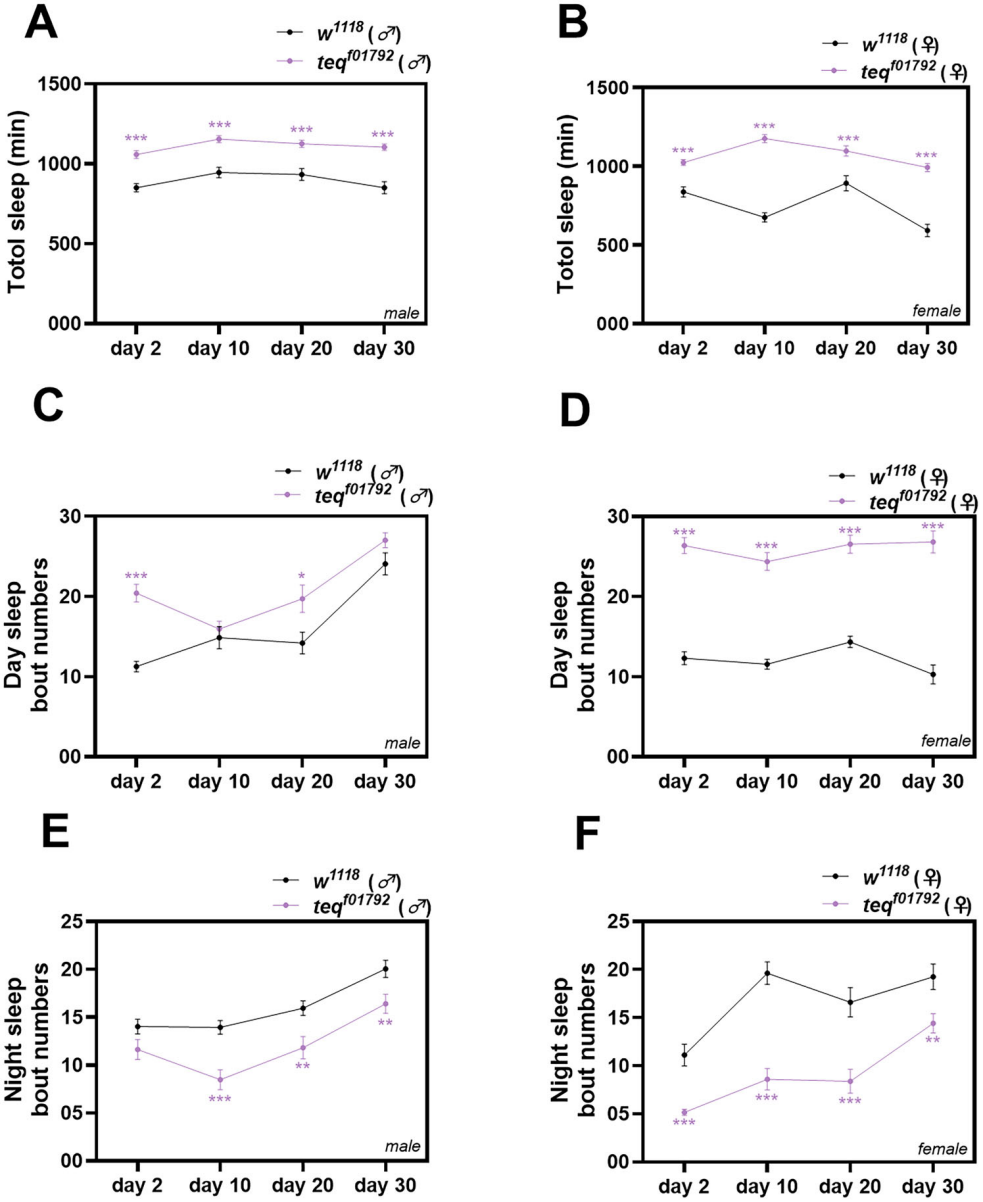
Mutation of *tequila* gene leads to an increase in sleep fragmentation at an early age in flies. ***A***, ***B***, Total sleep duration in minutes of male and female *w^1118^* and *tequila^f01792^* flies across four different ages, namely, 2, 10, 20, and 30 d old. An increase in total sleep is observed in *tequila^f01792^* flies across all ages. In Day 20, *tequila^f01792^* female the sample size of flies was 20. ***C***, ***D***, Quantified average number of sleep bouts in the day for male and female *w^1118^* and *tequila^f01792^* flies across four different ages, namely, 2, 10, 20, and 30 d old. ***E***, ***F***, Quantified average number of sleep episodes in the night for male and female *w^1118^* and *tequila^f01792^* flies across four different ages, namely, 2, 10, 20, and 30 d old.

Sleep is a very dynamic process and is known to change with age (He and Jasper, 2014). With aging, sleep quality declines, resulting in increased sleep bout numbers and decreased mean sleep bout duration (Koh et al., 2006). Here, we have analyzed sleep quality of different age groups in *tequila^f01792^* flies. In all the age groups analyzed, a significant increase in total sleep was observed in both male and female *tequila^f01792^* flies, consistent with our previous results obtained with 2-d-old flies [Fig. 2*A*,*B*; *w*^*1118(*♂*)*^ (2-d-old) vs *tequila*^*f01792 (*♂*)*^ *t*_(61)_ = 5.628, *p* < 0.001; *w*^*1118(*♂*)*^ (10-d-old) vs *tequila*^*f01792 (*♂*)*^ *t*_(49)_ = 5.314, *p* < 0.001; *w*^*1118(*♂*)*^ (20-d-old) vs *tequila*^*f01792 (*♂*)*^ *t*_(46)_ = 3.236, *p* < 0.01; *w*^*1118(*♂*)*^ (30-d-old) vs *tequila*^*f01792 (*♂*)*^ *t*_(53)_ = 5.488, *p* < 0.001); *w*^*1118(*♀*)*^ (2-d-old) vs *tequila*^*f01792 (*♀*)*^ *t*_(53)_ = 4.654, *p* < 0.001; *w*^*1118(*♀*)*^ (10-d-old) vs *tequila*^*f01792 (*♀*)*^ *t*_(59)_ = 13.06, *p* < 0.001; *w*^*1118 (*♀*)*^ (20-d-old) vs *tequila*^*f01792 (*♀*)*^ *t*_(46)_ = 3.236, *p* = 0.002; *w*^*1118 (*♀*)*^ (30-d-old) vs *tequila*^*f01792 (*♀*)*^ *t*_(49)_ = 8.495, *p* < 0.001, unpaired *t* test]. Next, we wanted to verify if the flies of all ages showed sleep fragmentation during the daytime. To understand this, we quantified the number of sleep bouts with respect to the light and dark phase of the LD cycle. In *tequila^f01792^* male flies, we observed an increasing trend in sleep bout numbers in all ages when compared with the control. A significant increase in daytime sleep bouts was observed in flies aged 2 and 20 when compared with the control [Fig. 2*C*; *w*^*1118(*♂*)*^ (2-d-old) vs *tequila*^*f01792 (*♂*)*^ *t*_(61)_ = 7.041, *p* < 0.001; *w*^*1118(*♂*)*^ (20-d-old) vs *tequila*^*f01792 (*♂*)*^ *t*_(48)_ = 2.420, *p* = 0.02, unpaired *t* test]. However, the increase did not reach a statistically significant level for 10- and 30-d-old flies. On the other hand, female *tequila*^*f01792 (*♀*)*^ flies displayed a significant increase in daytime sleep bout numbers at all ages compared with the control [Fig. 2*D*; *w*^*1118(*♀*)*^ (2-d-old) vs *tequila*^*f01792 (*♀*)*^ *t*_(52)_ = 10.97, *p* < 0.001; *w*^*1118(*♀*)*^ (10-d-old) vs *tequila*^*f01792 (*♀*)*^ *t*_(60)_ = 10.16, *p* < 0.001; *w*^*1118 (*♀*)*^ (20-d-old) vs *tequila*^*f01792 (*♀*)*^ *t*_(52)_ = 9.034, *p* < 0.001; *w*^*1118 (*♀*)*^ (30-d-old) vs *tequila*^*f01792 (*♀*)*^ *t*_(50) _= 9.15 *p* < 0.001].

Further, we verified if the flies of all ages showed increased sleep consolidation in the night phase as observed in younger flies. In male flies, we observed a significant decrease in sleep bout numbers in all ages except 2-d-old male flies [Fig. 2*E*; *w*^*1118(*♂*)*^ (10-d-old) vs *tequila*^*f01792 (*♂*)*^ *t*_(49)_ = 4.376, *p* < 0.001; *w*^*1118(*♂*)*^ (20-d-old) vs *tequila*^*f01792 (*♂*)*^ *t*_(48)_ = 2.762, *p* < 0.008; *w*^*1118(*♂*)*^ (30-d-old) vs *tequila*^*f01792 (*♂*)*^ = *t*_(53)_ = 2.708, *p* < 0.009, unpaired *t* test]. In female flies, a significant decrease in the number of nighttime sleep bouts was observed in *tequila^f01792^* flies in all the four age groups compared with the control [Fig. 2*F*; *w*^*1118(*♀*)*^ (2-d-old) vs *tequila*^*f01792 (*♀*)*^ *t*_(51)_ = 4.652, *p* < 0.001, unpaired *t* test; *w*^*1118(*♀*)*^ (10-d-old) vs *tequila*^*f01792 (*♀*)*^ *t*_(60)_ = 6.833, *p* < 0.001, unpaired *t* test; *w*^*1118 (*♀*)*^ (20-d-old) vs *tequila*^*f01792 (*♀*)*^ *p* < 0.001, Mann–Whitney test; *w*^*1118 (*♀*)*^ (30-d-old) vs *tequila*^*f01792 (*♀*)*^ *t*_(50)_ = 2.885, *p* < 0.006, unpaired *t* test].

**Figure 3.**
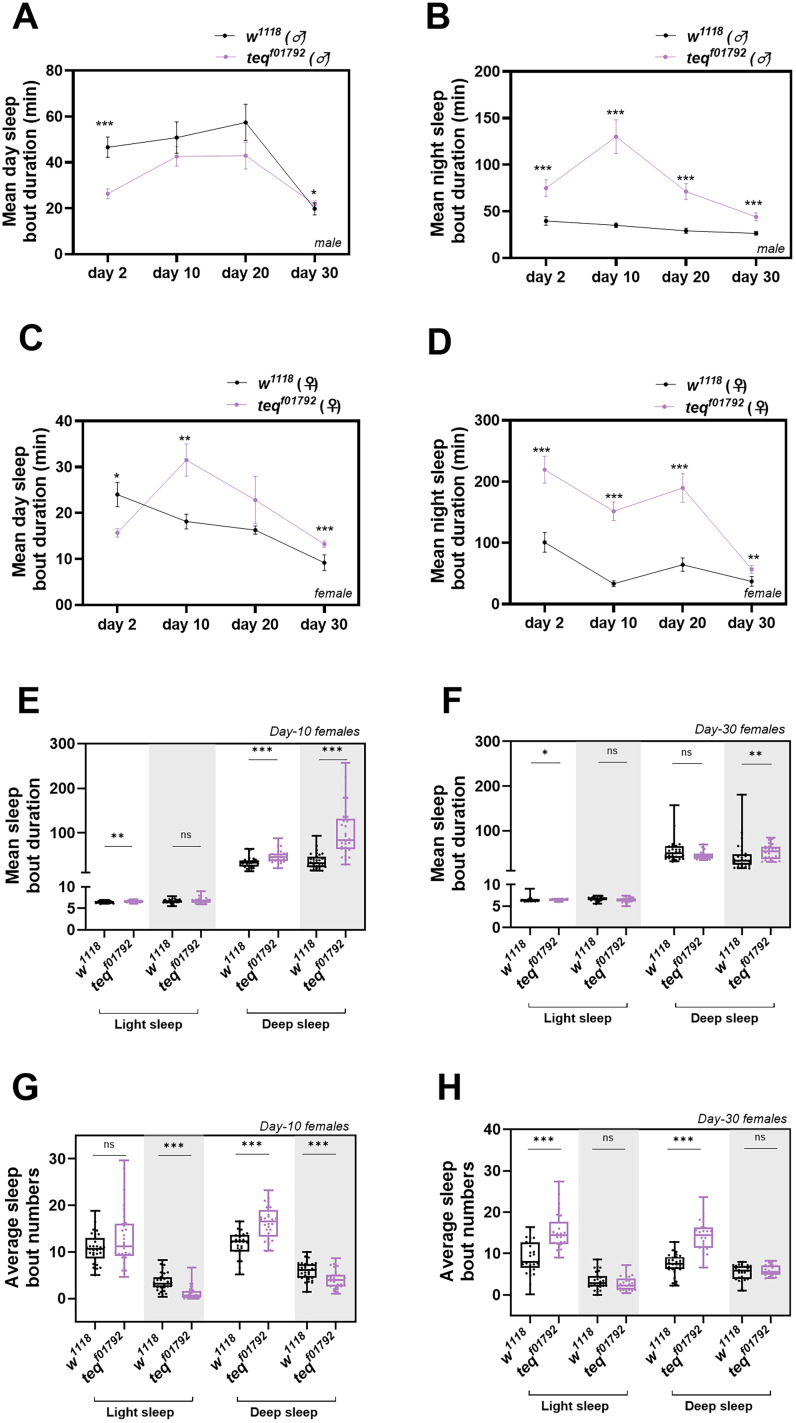
Mutation of *tequila* gene shows sexual dimorphism in sleep fragmentation with increasing age flies. ***A***, Quantified daytime mean sleep bout duration in minutes for 2-, 10-, 20-, and 30-d-old *w^1118^* and *tequila^f01792^* male flies, respectively. ***B***, Quantified nighttime mean sleep bout duration in minutes for 2-, 10-, 20-, and 30-day-old *w^1118^* and *tequila^f01792^* male flies, respectively. ***C***, Quantified daytime mean sleep bout duration in minutes for 2-, 10-, 20-, and 30-d-old *w^1118^* and *tequila^f01792^* female flies, respectively. ***D***, Quantified nighttime mean sleep bout duration in minutes for 2-, 10-, 20-, and 30-d-old *w^1118^* and *tequila^f01792^* female flies, respectively. ***E***, Quantified day and nighttime light and deep sleep bout duration in minutes for 10-d-old female *w^1118^* and *tequila^f01792^* flies. ***F***, Quantified day and nighttime light and deep sleep bout duration in minutes for 30-d-old female *w^1118^* and *tequila^f01792^* flies. ***G***, Day and night time light and deep sleep bout numbers of 10-d-old *w^1118^* and *tequila^f01792^* female flies. ***H***, Day and night time light and deep sleep bout numbers of 30-d-old *w^1118^* and *tequila^f01792^* female flies.

We further quantified the sleep bout duration across ages in both male and female flies. In male flies a significant decrease in daytime bout duration was observed in 2- and 30-d-old *tequila^f01792^* flies. No significant decrease but a decreasing trend was observed in 10- and 20-d-old *tequila^f01792^* flies [Fig. 3*A*; *w*^*1118(*♂*)*^ (2-d-old) vs *tequila*^*f01792 (*♂*)*^ *t*_(59)_ = 4.213 *p* < 0.001, unpaired *t* test; *w*^*1118(*♂*)*^ (30-d-old) vs *tequila*^*f01792 (*♂*)*^ *p* = 0.04, Mann–Whitney test]. During the night, *tequila*^f01792^ flies of all ages showed an increase in sleep bout duration when compared with the control flies [Fig. 3*B*; *w*^*1118(*♂*)*^ (2-d-old) vs *tequila*^*f01792 (*♂*)*^ *p* < 0.001; *w*^*1118(*♂*)*^ (10-d-old) vs *tequila*^*f01792 (*♂*)*^ *p* < 0.001; *w*^*1118(*♂*)*^ (20-d-old) vs *tequila*^*f01792 (*♂*)*^ *p* < 0.001; *w*^*1118(*♂*)*^ (30-d-old) vs *tequila*^*f01792 (*♂*)*^ *p* < 0.001, Mann–Whitney test]. In females, a decrease in daytime bout duration was observed only in young 2-d-old *tequila^f01792^* flies [Fig. 3*C*; *w*^*1118(*♀*)*^ (2-d-old) vs *tequila*^*f01792 (*♀*)*^ *p* = 0.03, Mann–Whitney test]. However, in 10- and 30-d-old *tequila^f01792^* flies, a significant increase in the bout duration was observed during the daytime when compared with the control flies [Fig. 3*C*; *w*^*1118(*♀*)*^ (10-d-old) vs *tequila*^*f01792 (*♀*)*^ *p* = 0.001; *w*^*1118(*♀*)*^ (30-d-old) vs *tequila*^*f01792 (*♀*)*^ *p* < 0.001, Mann–Whitney test]. In the nighttime, a significant increase in sleep bout duration was observed in *tequila*^f01792^ female flies of all ages when compared with the control flies [Fig. 3*D*; *w*^*1118(*♀*)*^ (2-d-old) vs *tequila*^*f01792 (*♀*)*^ *p* < 0.001; *w*^*1118(*♀*)*^ (10-d-old) vs *tequila*^*f01792 (*♀*)*^ *p* < 0.001; *w*^*1118(*♀*)*^ (20-d-old) vs *tequila*^*f01792 (*♀*)*^ *p* < 0.001; *w*^*1118(*♀*)*^ (30-day-old) vs *tequila*^*f01792 (*♀*)*^ *p* = 0.002, Mann–Whitney test].

As we observed an increase in bout duration and bout numbers during the daytime in female 10- and 30-d-old flies, we further analyzed their light and deep sleep bout duration during the daytime and nighttime. In 10-d-old female flies, we observed a significant increase in the light and deep sleep bout duration during the daytime when compared with the control flies [Fig. 3*E*; *w*^*1118(*♀*)*^ (10-d-old, light sleep) vs *tequila*^*f01792 (*♀*)*^ *t*_(60)_ = 2.996, *p* = 0.004; *w*^*1118(*♀*)*^ (10-d-old, deep sleep) vs *tequila*^*f01792 (*♀*)*^ *t*_(60)_ = 4.336, *p* < 0.001, Student's *t* test]. In the 30-d-old flies, only a significant increase in the light sleep bout duration was observed [Fig. 3*F*; *w*^*1118(*♀*)*^ (30-d-old, light sleep) vs *tequila*^*f01792 (*♀*)*^, *p* = 0.02, Mann–Whitney test]. However, during the nighttime both 10- and 30-d-old flies showed increased deep sleep bout duration [Fig. 3*E*,*F*; *w*^*1118(*♀*)*^ (10-d-old, deep sleep) vs *tequila*^*f01792 (*♀*)*^ *p* < 0.001; *w*^*1118(*♀*)*^ (30-d-old, deep sleep) vs *tequila*^*f01792 (*♀*)*^ *p* = 0.06, Mann–Whitney test]. This resulted in an overall increase in the sleep bout duration during both day and night in 10- and 30-d-old female flies.

Next, we also quantified their light and deep sleep bout numbers across day and night. In 10- and 30-d-old *tequila*^*f01792 (*♀*)*^ female flies during the daytime, an increase in deep sleep bout numbers was observed when compared with the control [Fig. 3*G*,*H*; *w*^*1118(*♀*)*^ (10-d-old, deep sleep) vs *tequila*^*f01792 (*♀*)*^ *t*_(60)_ = 5.867, *p* < 0.001; *w*^*1118(*♀*)*^ (30-d-old, deep sleep) vs *tequila*^*f01792 (*♀*)*^ *t*_(60)_ = 7.956, *p* < 0.001, Student's *t* test]. Apart from this, 30-d-old flies also showed an increase in light sleep bout numbers [Fig. 3*H*; *w*^*1118(*♀*)*^ (30-d-old, deep sleep) vs *tequila*^*f01792 (*♀*)*^ *p* < 0.001, Mann–Whitney test]. During the nighttime, a decrease in light and deep sleep bout numbers was observed only in 10-d-old *tequila^f01792^* flies when compared with the control [Fig. 3*G*; *w*^*1118(*♀*)*^ (10-d-old, light sleep) vs *tequila*^*f01792 (*♀*)*^, *p* < 0.001, Mann–Whitney test; *w*^*1118(*♀*)*^ (10-d-old, deep sleep) vs *tequila*^*f01792 (*♀*)*^ *t*_(60)_ = 3.844, *p* < 0.001, Student's *t* test]. These results suggest that mutation in the *tequila* leads to sleep fragmentation at a very early age in male flies. Additionally, the mutation has age-dependent and sex-specific differential effects on daytime sleep and sleep consolidation, with female flies showing differential sleep patterns compared with their male counterparts.

### Pharmacological induction of sleep, using GABA agonist rescues sleep quality in male flies

**Figure 4.**
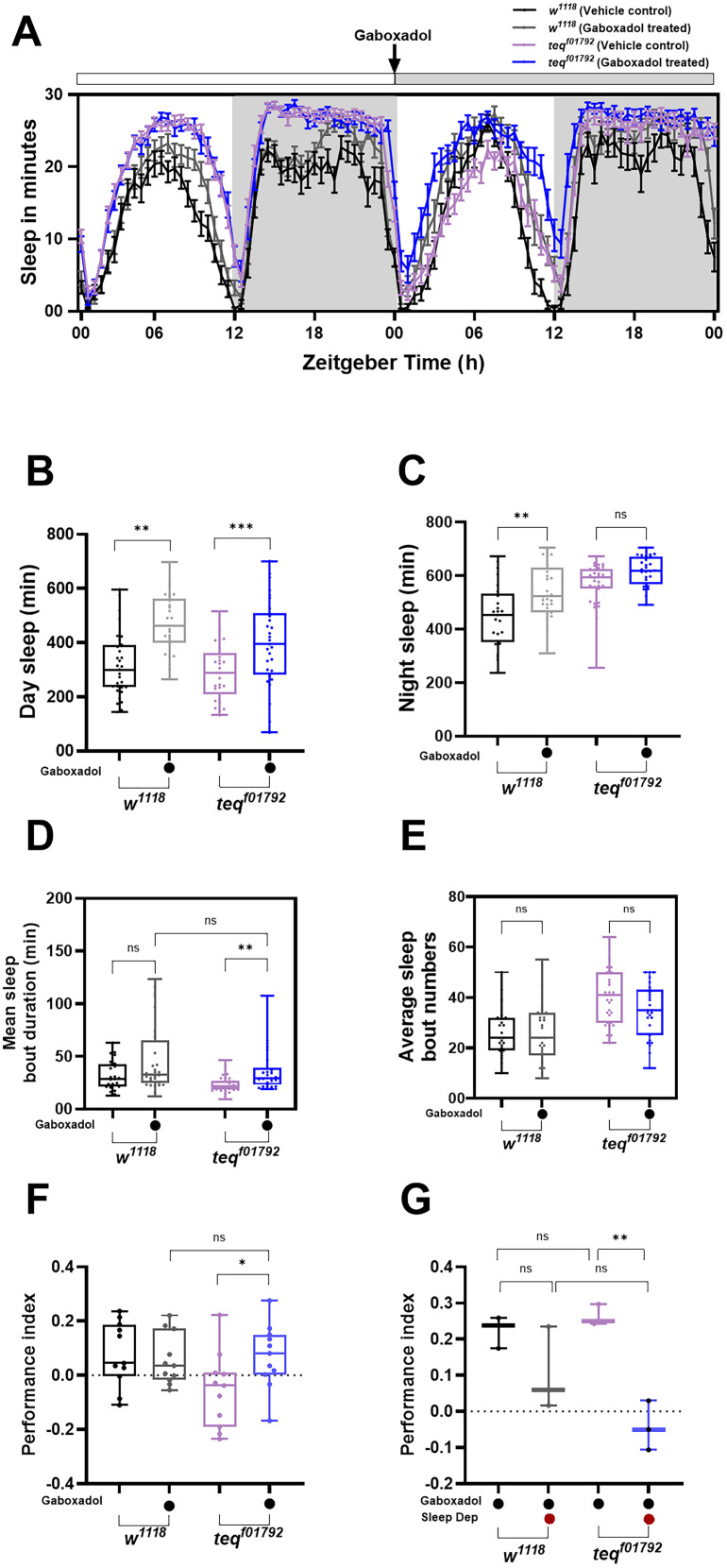
Pharmacological induction of sleep, using GABA-A agonist, rescues sleep quality in male flies. ***A***, Sleep profile of 2 consecutive days of *w^1118^* and *tequila^f01792^* flies. The first 24 h depicts the baseline sleep of *w^1118^* and *tequila^f01792^* flies. After 24 h just before the lights on, the flies were flipped to either media containing 0.1 mg/ml THIP or their vehicle control (MilliQ), and their sleep was recorded for the next 24 h. The gray shaded bar on top of the graph represents the duration of THIP treatment. ***B***, Quantified day sleep in minutes of *w^1118^* and *tequila^f01792^* flies before and after THIP treatment. An increase in day sleep was observed in both control and experimental flies treated with THIP. ***C***, Quantified night sleep in minutes of *w^1118^* and *tequila^f01792^* flies before and after THIP treatment. An increase in sleep was observed only in the control flies after drug treatment. ***D***, Quantified mean sleep bout duration of *w^1118^* and *tequila^f01792^* flies before and after THIP treatment. An increase in sleep bout duration was observed in both control and experimental flies post-drug treatment. ***E***, Average sleep bout number of *w^1118^* and *tequila^f01792^* flies before and after THIP treatment. ***F***, Performance index for pavlovian appetitive olfactory memory of *w^1118^* and *tequila^f01792^* flies before and after THIP treatment. *tequila^f01792^* flies showed an increase in the performance index post 0.1 mg/ml of THIP ingestion. ***G***, Performance index of *w^1118^* and *tequila^f01792^* flies who underwent mechanical sleep deprivation (sleep dep) along with 0.1 mg/ml of THIP treatment. The presence of sleep deprivation along with the drug does not improve the memory indicating it to be due to the effect of sleep and not the ingestion of the drug. Extended Data Figure 4-1 supports this figure.

Our results so far have shown that *tequila* mutant flies have a defect in sleep quality only during the daytime. Our next aim was to induce sleep and assess whether it could rescue sleep fragmentation. To induce sleep we used THIP, the GABA-A receptor agonist (Dissel et al., 2015). It has been previously observed that THIP induction leads to increased total sleep in flies (Dissel et al., 2015). To verify the effect of THIP on sleep, 2-d-old *w^1118^* flies were transferred to food containing 0.1 mg/ml of THIP, and their sleep was recorded for 24 h (Extended Data Fig. 4-1*A*). As previously reported, we were able to induce sleep upon THIP induction which led to increased day and night sleep (Extended Data Fig. 4-1*A–C*). Post verifying that THIP induction leads to increased total sleep, we provided THIP to *tequila^f01792^* flies to induce sleep. Both the control and *tequila^f01792^* flies treated with THIP showed an increase in day sleep when compared with their vehicle control fed flies [Fig. 4*A*,*B*; *w^1118^* vs *w^1118^* *+* THIP (day sleep) *p* = 0.008; *tequila^f01792^* vs *tequila^f01792^* + THIP (day sleep) *p* < 0.001, ANOVA followed by Tukey's multiple comparison]. In control flies an increase in night sleep was observed, but no changes in the night sleep were observed in *tequila^f01792^* when compared with the vehicle control flies [Fig. 4*A*,*C*; *w^1118^* vs *w^1118^* *+* THIP (night sleep) *p* = 0.002; *w^1118^* vs *tequila^f01792^* (night sleep) *p* < 0.001; ANOVA followed by Tukey's multiple comparison]. Although there were no changes in sleep quality in control flies upon THIP treatment, *tequila^f01792^* flies partially rescued sleep quality following THIP treatment. The total mean sleep bout duration in *tequila^f01792^* flies was significantly higher when compared with their respective vehicle control indicating better sleep consolidation [Fig. 4*D*; *tequila^f01792^* vs *tequila^f01792^* + THIP (mean sleep bout duration) *p* < 0.01, Kruskal–Wallis followed by Dunn's multiple comparisons]. Although sleep bout numbers were decreased in these flies compared with the vehicle control, this difference did not reach a statistically significant level (Fig. 4*E*). Thus, we conclude that THIP treatment is able to partially rescue the sleep quality in *tequila^f0179^*^*2*^ mutant flies.

10.1523/ENEURO.0566-24.2025.f4.1 Figure 4.1 Pharmacological induction of sleep, using GABA agonist, rescues sleep quality in *w^1118^* male flies (A) Sleep profile of *w^1118^* flies with and without THIP treatment. The first 24hr depicts the baseline sleep of *w^1118^* flies. After 24 hr just before the lights-on the flies were flipped to either media containing 0.1  mg/mL THIP or their vehicle control (MilliQ) and their sleep was recorded for the next 24 hr. (B) Quantified day-sleep of *w^1118^* flies before and after THIP treatment. (C) Quantified night-sleep of *w^1118^* flies before and after THIP treatment. An increase in sleep was observed in THIP treated *w^1118^* flies. (D) Performance index of *w^1118^* and *tequila ^f01792^* flies subjected to mechanical sleep deprivation combined with 0.1  mg/mL of THIP treatment. Download Figure 4.1, DOCX file.

### Inducing sleep using GABA agonist rescues LTM impairment in *tequila ^f01792^* male flies

Upon verifying that THIP was able to rescue sleep fragmentation in *tequila^f01792^* mutant flies, our next goal was to test whether we could rescue LTM impairment observed in *tequila^f01792^* flies with the same treatment. To do this, we followed the same protocol and provided flies with 48 h of THIP treatment before the starvation phase prior to conducting the olfactory memory training. Upon induction of sleep through THIP, *tequila^f01792^* flies performed better in appetitive odor-associated olfactory memory when compared with the vehicle control flies (Fig. 4*F*; *tequila*^*f01792 (*♂*) (THIP)*^ vs *tequila*^*f01792 (*♂*) (VC)*^ *t*_(20)_ = 2.320, *p* = 0.03, unpaired *t* test). Furthermore, the performance index of control and *tequila^f01792^* flies after sleep induction did not differ significantly indicating the restoration of memory (Fig. 4*F*). These results indicate that the *tequila* gene plays a role in LTM and most probably by influencing sleep-dependent memory consolidation pathways. To ensure that the memory improvement observed was not due to THIP ingestion but due to the sleep induced upon drug ingestion, we conducted sleep deprivation experiments in the presence of the drug. Unlike flies treated with THIP, flies fed with THIP which underwent a simultaneous mechanical sleep deprivation did not show any improvement in memory formation (Fig. 4*G*, Extended data Fig. 4-1*D*; *tequila^f01792^* vs *tequila^f01792^* + THIP *t*_(4)_ = 7.096, *p* < 0.002, unpaired *t* test followed by Tukey's multiple comparison). Thus, it is not the effect of the drug but the sleep induced by the drug which helps in rescuing LTM consolidation upon deletion of the *tequila* gene in flies.

### Downregulation of *tequila* in the MB does affect LTM but not the sleep quality in male flies

**Figure 5.**
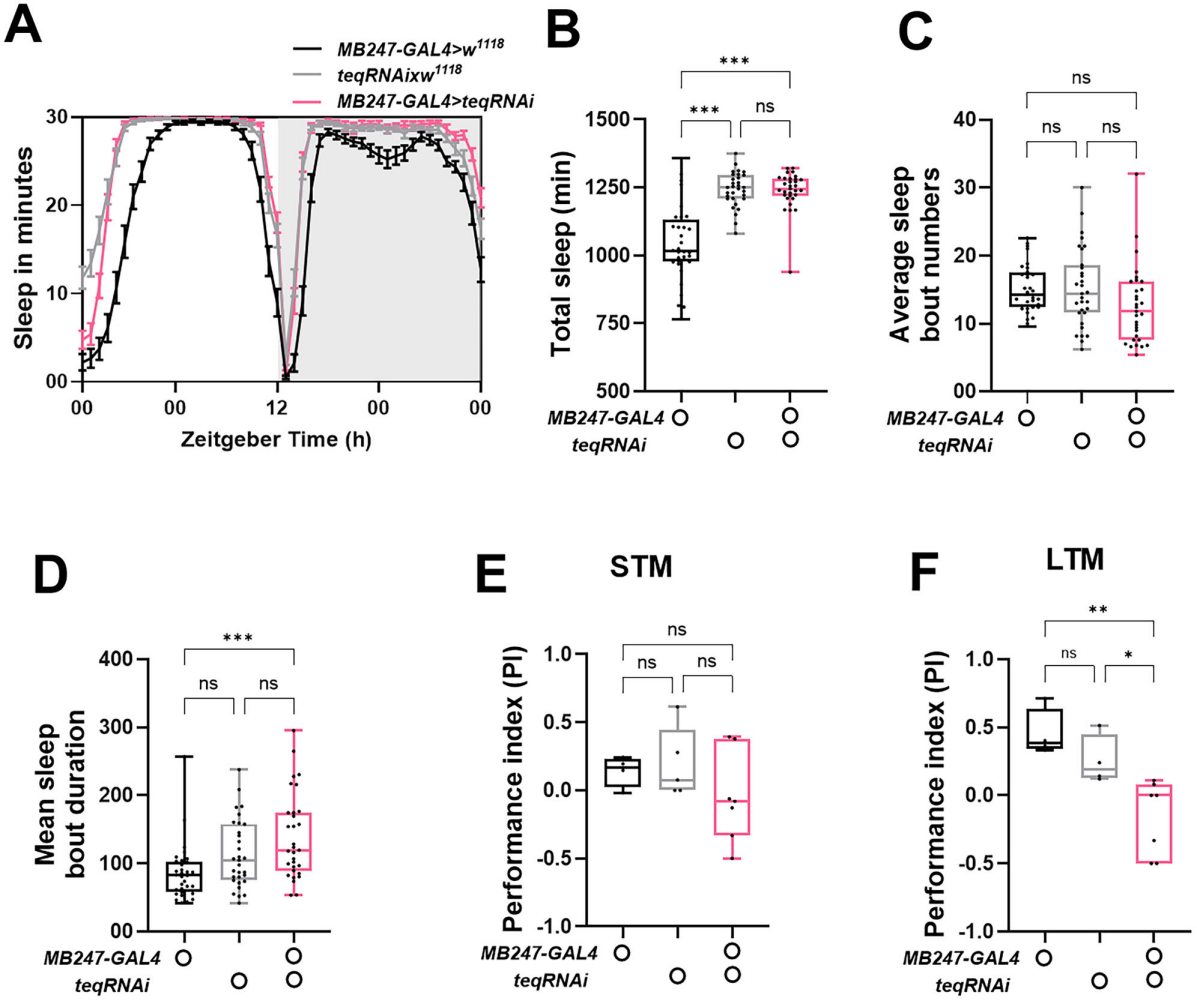
Downregulation of *tequila* in the MB does not affect sleep quality in male flies. ***A***, Sleep in minutes for every 30 min over a period of 24 h averaged across 5 d under LD for 2-d-old *MB247-Gal4* *>* *w^1118^*, *w^1118^* *×* *teqRNAi, MB247-Gal4* *>* *teqRNAi* flies. ***B***, Total sleep duration of *MB247-Gal4* *>* *w^1118^*, *w^1118^* *×* *teqRNAi, MB247-Gal4* *>* *teqRNAi* flies. No significant difference in sleep duration was observed. ***C***, Average sleep bout numbers of *MB247-Gal4* *>* *w^1118^*, *w^1118^* *×* *teqRNAi, MB247-Gal4* *>* *teqRNAi* flies across 5 d. No significant difference was observed. ***D***, Mean sleep bout duration of *MB247-Gal4* *>* *w^1118^*, *w^1118^* *×* *teqRNAi, MB247-Gal4* *>* *teqRNAi* of 2-d-old flies averaged across 5 d. No significant difference in sleep duration was observed. ***E***, STM performance index using operant appetitive olfactory memory of *MB247-Gal4* *>* *w^1118^*, *w^1118^* *×* *teqRNAi, MB247-Gal4* *>* *teqRNAi* of 2-d-old flies. ***F***, LTM performance index using operant appetitive olfactory memory of *MB247-Gal4* *>* *w^1118^*, *w^1118^* *×* *teqRNAi, MB247-Gal4* *>* *teqRNAi* of 2-d-old flies.

Previously it was shown that MB neurons are involved in *tequila* mediated LTM formation induced by aversive olfactory conditioning (Didelot et al., 2006)*.* We wanted to check if MB neurons were responsible for *tequila (teq)* mediated sleep fragmentation as well. To do this we downregulated *tequila* gene specifically in the MB using the *MB247-Gal4.* We analyzed their total sleep, sleep bout numbers, and sleep duration, and we did not observe any significant differences in either sleep quantity or quality when compared with both the controls (Fig. 5*A–D*). Next, we measured their appetitive olfactory memory using the Y-maze. Downregulation of *teq* in the MB resulted in a decrease in appetitive olfactory LTM but not any STM defects (Fig. 5*E*,*F*; *MB247-Gal4* *>* *w^1118^* vs *MB247-Gal4* *>* *teqRNAi p* = 0.003; *w^1118^* *×* *teqRNAi* vs *MB247-Gal4* *>* *teqRNAi p* = 0.03, ANOVA followed by Tukey's multiple comparisons). These results suggest that *tequila* expression in the MB is involved in mediating LTM induced by appetitive olfactory conditioning but the effect of *tequila* on sleep quality is not mediated through the MB neurons.

10.1523/ENEURO.0566-24.2025.d1 Raw data This manuscript contains extended data, including the raw data for the figures. Figure 1- Raw data and statistical tests for Figure 1; Figure 1-1- Raw data and statistical tests for extended data of Figure 1; Figure 2- Raw data and statistical tests for Figure 2; Figure 3- Raw data and statistical tests for Figure 3, Figure 4- Raw data and statistical tests for Figure 4; Figure 4-1- Raw data and statistical tests for extended data of Figure 4; Figure 5- Raw data and statistical tests for Figure 5; Light and deep sleep fragmentation analysis in Drosophila- Python code and readme file used for analysis. Download Raw data, ZIP file.

## Discussion

While the physiological mechanisms that govern memory consolidation and retrieval are complex and difficult to decipher, numerous studies highlight the critical role of sleep in memory consolidation (Margulies et al., 2005; Chouhan et al., 2021; Chouhan and Sehgal, 2022; Lei et al., 2022; Marquand et al., 2023). Previous studies have shown that sleep and memory processes are correlated, not only does sleep facilitate memory consolidation (Bushey et al., 2007; Donlea et al., 2011; Dissel et al., 2015; Chouhan et al., 2021), but learning also increases the drive for sleep (Lei et al., 2022). In our study, for the first time, we show the importance of sleep quality maintenance in the consolidation of long-term appetitive memory in *Drosophila*. Mutation of the gene *tequila*, a serine protease, leads to increased sleep fragmentation and LTM impairment in flies.

We indeed observed that *tequila^f01792^* flies showcase a very peculiar yet recurring sleep pattern. It displays increased sleep fragmentation during the light phase and a compensatory increase in sleep consolidation during the dark phase of LD when compared with controls. According to the two-process sleep model developed by Borbély (1982), sleep pressure is controlled by the circadian clock (C-process) and the sleep homeostatic component (S-process; Borbély et al., 2016; Abhilash and Shafer, 2024). The S-process determines the discharge and the build-up of the sleep pressure which guides the sleep–wake cycle. During sleep deprivation, there is a build-up in sleep pressure which extends the sleep onset, leading to increased sleep during the subsequent recovery day (Borbély et al., 2016; Beckwith and French, 2019). As we observed a significant fragmentation of sleep during the day, we hypothesize the internal sleep drive to be altered due to synaptic plasticity defects leading to increased sleep during the dark phase of the LD cycle (Fig. 1*C*,*D*).

Notably, this sleep fragmentation phenotype is not uniformly present across age and sex. While male *tequila^f01792^* flies exhibited sleep fragmentation at an early age, some sexually dimorphic differences were observed, such as female *tequila^f01792^* flies exhibiting increased daytime sleep bout numbers and an increase in daytime sleep duration. This indicates inherent sex-specific differences in sleep regulation (Wu et al., 2018). However further empirical evidence is required to dissect these sex-specific daytime sleep quality and quantity differences observed in *tequila^f01792^* mutant flies. With increasing age, sleep becomes more fragmented in *Drosophila* (Koh et al., 2006). Male *tequila^f01792^* flies showed sleep fragmentation at an earlier age compared with control flies suggesting that *tequila*'s role in sleep quality may be more prominent in early adulthood. This sleep fragmentation observed at early age did not intensify with increasing age in *tequila^f01792^* flies. However daytime sleep fragmentation probably impairs the internal sleep drive, leading to a consistent nighttime sleep consolidation across all ages in tequila mutant flies.

Neuromodulators such as insulin are known to modulate sleep drive. Previous studies have shown that impaired insulin signaling is associated with sleep disturbances (Metaxakis et al., 2014). Furthermore, in humans it was shown that acute insulin administration into the central nerve canal through intranasal ingestion improves memory consolidation (Born et al., 2002; Feld et al., 2016). The improved memory observed upon insulin ingestion was attributed to its function in the hippocampus for long-term potentiation (van der Heide et al., 2005; Moult and Harvey, 2008; Feld et al., 2016). In *Drosophila* as well as Insulin-Like Peptides (dILP)-2, 3, and 5, triple mutant fly exhibits sleep impairment with reduced daytime sleep and increased night sleep similar to the hypomorphic *tequila* mutants (Metaxakis et al., 2014). Apart from this, *tequila^f01792^* flies have increased life span, which has been attributed to decreased insulin signaling (Huang et al., 2015). This study speculated that *tequila* might act as a neurotrypsin to the DILP2 protein. In the absence of the neurotrypsin, insulin signaling may reduce and thus potentially leads to an increased life span. Given that deletion of dILP in flies affects daytime sleep duration in flies, and *tequila* mutants also showed decreased insulin signaling, the sleep fragmentation observed in tequila mutant flies could be due to impaired insulin signaling.

Our results indicate that by rescuing sleep consolidation using a GABA-A agonist, we were also able to alleviate memory impairment. When the flies were sleep deprived in the presence of the drug, no improvement in memory was observed, indicating that the enhancement in memory is probably due to improved sleep rather than a direct drug effect. However, both *tequila* and GABA-A receptors are expressed in MB (Didelot et al., 2006; Liu et al., 2007), but whether there is any functional interaction between them remains unclear. If *tequila* mutations reduce GABA-A receptor signaling in the MB, THIP administration may restore this signaling, potentially rescuing the LTM deficits. Therefore, it remains important to determine whether the sleep fragmentation improvement observed in *tequila* mutant is due to the restoration of GABA-A signaling in the MB. It is also crucial to identify the specific neurons responsible for mediating sleep fragmentation in *tequila* mutants.

Previously, it was shown that the MB is involved in *tequila* mediated LTP formation in flies (Didelot et al., 2006). However, upon downregulation of *tequila* specifically in the MB, there were no sleep quality defects observed in these flies. Downregulating tequila specifically in the MB did not recapitulate the sleep quality defects observed in hypomorphic mutants, indicating that sleep fragmentation is probably mediated through neuronal populations outside the MB. Further studies are required to identify the neuronal circuits involved in this sleep fragmentation.

In the present study, we used the *tequila^f01792^* mutant, which is currently the only available mutant for the *tequila* gene. We were unable to validate our findings using additional *tequila* mutant lines. Furthermore, genetic rescue through the expression of a functional copy of the gene in the mutant background is required to validate our findings, and future studies will be directed toward addressing this aspect. Although THIP is a widely used drug for artificially inducing sleep, further studies are needed to confirm whether the improvement in sleep fragmentation indeed enhances memory. To this end, future studies will explore the effects of other sleep-promoting neurotransmitters, such as serotonin (5-HT), as well as serotonin reuptake inhibitors and genetic tools for sleep induction, to determine whether they similarly alleviate sleep fragmentation and improve memory.

To conclude, our study demonstrates for the first time the critical role of sleep quality in memory consolidation. The *tequila* gene plays an essential role in determining sleep consolidation in flies, and the mutation on this gene leads to increased daytime sleep fragmentation leading to impaired LTM in these flies.

## Synthesis

Reviewing Editor: Silvia Pagliardini, University of Alberta Decisions are customarily a result of the Reviewing Editor and the peer reviewers coming together and discussing their recommendations until a consensus is reached. When revisions are invited, a fact-based synthesis statement explaining their decision and outlining what is needed to prepare a revision will be listed below. The following reviewer(s) agreed to reveal their identity: François Rouyer, Takaomi Sakai. Note: If this manuscript was transferred from JNeurosci and a decision was made to accept the manuscript without peer review, a brief statement to this effect will instead be what is listed below.

Reviewer 1

This study by segu et al. analyzes sleep in tequila mutants, previously shown to have long term memory defects, and show that the mutants have sleep defects, particularly daytime sleep defects with an increase of the number of sleep bouts and a decrease of sleep bout duration (sleep fragmentation). Interestingly, treating flies with a sleep-promoting GABA-A agonist increases both sleep and memory defects, suggesting that daytime sleep fragmentation might be responsible for the memory deficit in the mutant.

The authors made significant improvements to the manuscript and answered the main points that were raised in my review. I nevertheless would recommend to include in the abstract the fact that the sleep effect is mainly observed in males.

Reviewer 2

The authors have shown, for the first time, that the tequila mutant flies exhibit sleep fragmentation. Young tequila mutant flies displayed significant disruptions in sleep continuity, and pharmacological experiments revealed that this fragmentation contributes to impaired long-term memory. Notably, this study is the first to demonstrate that the serine protease not only regulates sleep quality but also plays a role in sleep-dependent memory consolidation. These findings provide new insights into the role of sleep in Drosophila long-term memory.

The manuscript has been thoroughly revised from the previous version, and all of my concerns have been fully addressed. First, it is now clearly stated that the tequila mutant used in the experiments shares the genetic background of w1118, and that the experimental comparisons were made between the tequila mutant and the genetic control (w1118). The manuscript also provides a more detailed explanation of the sleep phenotype and sex differences. In addition, the authors have clarified their key claim: that reduced Tequila expression leads to daytime sleep fragmentation in young flies, which is believed to result in impaired long-term memory. Follow-up experiments further strengthen this conclusion. The authors show that mushroom body (MB)-specific tequila knockdown (KD) impairs long-term memory (LTM) in appetitive associative olfactory learning, while not affecting sleep. This finding suggests that the tequila-positive neurons involved in memory and those involved in sleep are distinct populations-adding further support to their argument. I have no major comments. Below,

I have noted a few minor typographical errors.

１）Delete the first word "are" in line 153.

２）Replace 'drosophila' with "Drosophila" in line 451.
