# Supplementary material for: Tequila, the Serine Protease, Is Involved in Sleep-Dependent Memory Consolidation in Drosophila
Source: eNeuro. 2025 Aug 27;12(8):ENEURO.0566-24.2025. doi: 10.1523/ENEURO.0566-24.2025 (PMC12501825; doi:10.1523/ENEURO.0566-24.2025)
Supplement: Figure 1-1 — tequila f01792 flies female flies display increased sleep bout numbers and increased bout duration. (A) Day and nighttime light and deep sleep bout numbers of 2-day-old w1118 and tequila f01792 female flies. An increase in the light and deep sleep bout numbers during the day-time was observed. (B) Day and nighttime light and deep sleep bout numbers of 2-day-old w1118 and tequila f01792 male flies. (C) Quantified total locomotor activity of 2-day old male and female w1118 and tequila f01792 flies respectively. A decrease in activity was observed in tequila f01792 flies. Download Figure 1-1, DOCX file. [file eneuro-12-ENEURO.0566-24.2025-s001.docx]

**
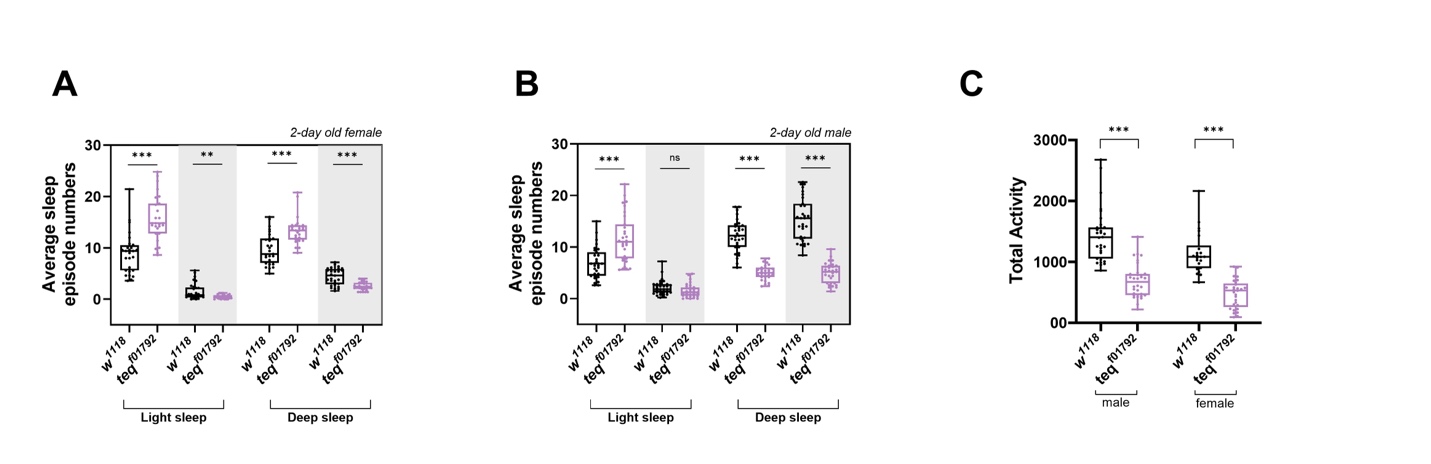
Figure 1-1.  *tequila ^f01792^* flies female flies display increased sleep bout numbers and increased bout duration.**

**(A) Day and night time light and deep sleep bout numbers of 2-day-old *w^1118^* and *tequila ^f01792^* female flies. An increase in the light and deep sleep bout numbers during the day-time was observed. (B) Day and night time light and deep sleep bout numbers of 2-day-old *w^1118^* and *tequila ^f01792^* male flies. An increase only in the light sleep bout numbers during the day-time was observed. (C) Quantified total locomotor activity of 2-day old male and female *w^1118^* and *tequila ^f01792^* flies respectively. A decrease in activity was observed in *tequila ^f01792^* flies.**
