# Supplementary material for: Tequila, the Serine Protease, Is Involved in Sleep-Dependent Memory Consolidation in Drosophila
Source: eNeuro. 2025 Aug 27;12(8):ENEURO.0566-24.2025. doi: 10.1523/ENEURO.0566-24.2025 (PMC12501825; doi:10.1523/ENEURO.0566-24.2025)
Supplement: Figure 4.1 — Pharmacological induction of sleep, using GABA agonist, rescues sleep quality in w1118 male flies (A) Sleep profile of w1118 flies with and without THIP treatment. The first 24hr depicts the baseline sleep of w1118 flies. After 24 hr just before the lights-on the flies were flipped to either media containing 0.1 mg/mL THIP or their vehicle control (MilliQ) and their sleep was recorded for the next 24 hr. (B) Quantified day-sleep of w1118 flies before and after THIP treatment. (C) Quantified night-sleep of w1118 flies before and after THIP treatment. An increase in sleep was observed in THIP treated w1118 flies. (D) Performance index of w1118 and tequila f01792 flies subjected to mechanical sleep deprivation combined with 0.1 mg/mL of THIP treatment. Download Figure 4.1, DOCX file. [file eneuro-12-ENEURO.0566-24.2025-s002.docx]

**
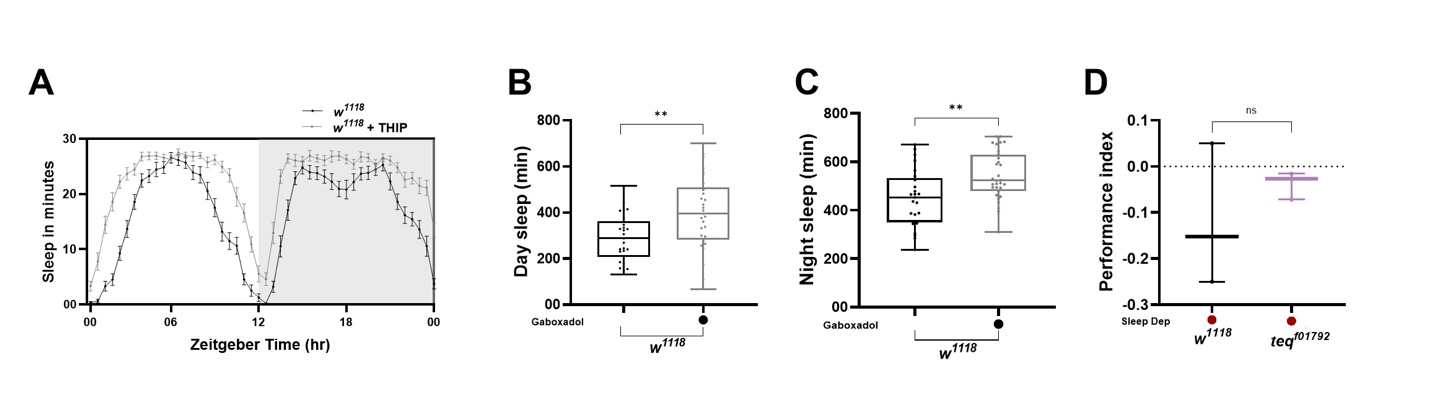
**

**Figure 4-1. Pharmacological induction of sleep, using GABA agonist, rescues sleep quality in *w^1118^* male flies**

**(A) Sleep profile of *w^1118^* flies with and without THIP treatment. The first 24hr depicts the baseline sleep of *w^1118^* flies. After 24 hr just before the lights-on the flies were flipped to either media containing 0.1 mg/mL THIP or their vehicle control (MilliQ) and their sleep was recorded for the next 24 hr. (B) Quantified day-sleep of *w^1118^* flies before and after THIP treatment. (C) Quantified night-sleep of *w^1118^* flies before and after THIP treatment. An increase in sleep was observed in THIP treated *w^1118^* flies. (D) Performance index of *w^1118^* and *tequila ^f01792^* flies subjected to mechanical sleep deprivation combined with 0.1mg/mL of THIP treatment.**
